# Correspondence

**Published:** 1872-12

**Authors:** Carl Proegler


### Correspondence.

EDITORS CHICAGO MEDICAL JOURNAL:

*Gentlemen*—In the November number of your valuable JOURNAL I find that Dr. Etheridge, of your place, reports from the *Vienna Medical Press*, August 18, a so-called victory of Stricker, or, more properly speaking, the Vienna school. That you may judge for yourselves, and on account of the appearance of a translation of "Stricker's Manual of Histology," I give you a translation of a correspondent, writing from Leipzig, the 7th of September, to the *Allgemeine Wiener Medical Zeitung:*

"Stricker wanted to show the results of his microscopical investigation on the inflammation of joints, and tried to base his theory on various preparations, consisting of tissues of the cornea rendered by traumatic influences artificially inflamed, and from these tissues Stricker wanted to show that they divide, reproduce, become amœboid, etc. But as microscopical authorities examined the preparations, serious doubts were expressed as to the correctness of Stricker's view. Especially did he fail to prove that the cornea cells, and the corneal tissue, underwent such transformations as he affirmed. The whole discovery made the impression of a doubtful, not clear, statement. Was the corneal tissue really changed, as Stricker affirmed? It was not proved beyond a doubt.

"Cohnheim treated the whole investigation in a very haughty, scornful manner, and offended, with his arrogance, the whole Vienna school. Even *Rindfleisch*, whose judgment is authoritative, was unable to give a favorable opinion about Stricker's investigations.

"Especially was the method used for injection not an exact one, and not at all satisfactory. With a more careful manipulation, the tissue of the cornea could have been shown to be intact, and with this assertion the whole theory of Stricker crumbles to pieces.

"This was the impression it made on the audience: if it had not been for Cohnheim's arrogance, Stricker's fiasco would have been more felt. Some of Stricker's disciples and friends, mostly military surgeons, tried to mask the fiasco by shouting and crying, and dispatched a telegram to Vienna, stating a victory for Stricker, only to throw sand in the eyes of the medical world; *but the learned men will, in spite of all this manœuvering, regard all the works of Stricker with distrust.* We Vienna men can only be sorry that we did not find a better representative of our school, having an abundance of great men who have done a good deal without boasting of it."

In all my German medical journals (and I keep about a dozen) Stricker is severely criticised, and we on this side of the water would do well not to trust too much in his investigations, as real authorities, like Virchow, Rindfleisch, etc., do not put any reliance in his works.

Respectfully yours,

DR. CARL PROEGLER.

